# Prevalence of cardiovascular risk factors in non-menopausal and postmenopausal inpatients with type 2 diabetes mellitus in China

**DOI:** 10.1186/s12902-019-0427-7

**Published:** 2019-10-11

**Authors:** Huanhuan Zhou, Chenghuan Zhang, Jingyu Ni, Xiaoyun Han

**Affiliations:** The First People’s Hospital of Changzhou, 185# Juqian Road, Changzhou, 213000 Jiangsu Province China

**Keywords:** Diabetes mellitus, Type 2, Menopause, Cardiovascular disease, Framingham risk score

## Abstract

**Background:**

To investigate the prevalence of cardiovascular disease (CVD) risk factors and assess the 10-year risk of CVD in non-menopausal and postmenopausal women with type 2 diabetes mellitus (T2DM).

**Methods:**

A total of 569 patients with T2DM at a Chinese tertiary hospital were investigated using the Framingham Risk Score (FRS). We evaluated the 10-year risk of CVD, clinical and menopause characteristics in all subjects.

**Results:**

Among the 569 diabetic patients, the incidence of smoking, dyslipidemia, hypertension, overweight or obesity, and nonalcoholic fatty liver disease (NAFLD) was 0.7, 36.2, 38.1 56.6 and 58.2%, respectively. The usage rate of hypoglycemic agents, antihypertensive agents, lipid modulators and antithrombotic drugs was 88.6, 78.3, 50.0 and 27.1%, respectively. However, only 1.2% of inpatients achieved the three target goals for the control of blood glucose (HbA_1c_ < 7%), blood pressure (systolic blood pressure < 130 mmHg, diastolic blood pressure < 80 mmHg), and blood lipids (total cholesterol < 174 mg/dL). The 10-year risk of CVD was (1.6 ± 1.5%) and tended to increase along with age (*F* = 27.726, *P* <  0.001). For all subjects (*n* = 569), multiple linear regression analysis showed that menopause (*β* = 0.275, *P* <  0.001), low-density lipoprotein cholesterol (LDL-C) (*β* = 0.212, *P* <  0.001), fasting plasma glucose (FPG) (*β* = 0.093, *P* = 0.018) and waist-to-hip-ratio (*β* = − 0.078, *P* = 0.047) were risk factors of 10-year risk of CVD, which may explain the variance of 14.3%. In the postmenopausal group (*n* = 397), LDL-C (*β* = 0.227, *P* <  0.001), FPG (*β* = 0.139, *P* = 0.003) and time since menopause (*β* = 0.230, *P* <  0.001) were found to be associated with CVD, which may explain the variance of 14.6%.

**Conclusion:**

The incidence of dyslipidmia, hypertension, overweight or obesity and NAFLD is high. The level of control of blood glucose, blood pressure, and blood lipids was found to be extremely low and the treatment status was not ideal. Besides menopause, LDL-C, FPG and time since menopause were found to be independent risk factors for the 10-year risk of CVD. Therefore, it is necessary to focus on comprehensive control of multiple risk factors, such as plasma glucose, blood pressure and serum lipid.

## Background

Cardiovascular disease (CVD) is a leading cause of mortality and morbidity worldwide [1]. The prevalence of CVD in China has increased rapidly since 2010, with stroke and ischemic heart disease accounting for most deaths [2] and is the first cause of death in Chinese women. However, in previous studies, compared to non-menopausal women, the incidence of CVD among postmenopausal women was found to be higher [3, 4]. The association between menopausal transition and the incidence of CVD is well known. For example, the increase in CVD risk is related to significant hormonal changes, especially estrogen deprivation at the time of menopause [4]. A decrease in oestradiol levels can lead to metabolic disorders, such as dyslipidemia, hypertension and increased central adiposity, which are considered to be cardiovascular risk factors [4–6].

As is well known, diabetes mellitus is a significant risk factor for developing CVD. The major pathological basis of CVD begins with endothelial damage or dysfunction [7, 8]. A review has suggested that the female sex hormone estrogen mediates the relative protection of premenopausal women against cardiovascular disease, compared with age-matched men [9]. However, these protective effects are absent in women with diabetes [10, 11]. Among diabetic patients, high glucose promotes the increase of reactive oxygen species formation, which may neutralize the vasorelaxant and anti-inflammatory effects of NO [12]. In hyperglycemia, the estrogen receptors α to estrogen receptors β ratio declined with the decreased secretion of estrogen modulation on endothelial estrogen receptor expression, which may lead to vascular injury [13]. Diabetes also changes the vascular tissue at the molecular level by enhancing oxidative stress and pro-inflammatory response, as well as advancing glycation end products and protein Kinase C activation, which together promote the development of CVD [14]. In addition, among menopausal patients, age at menopause, time since menopause, and total reproductive years (i.e., the duration between menarche and menopause) may determine the duration and level of exposure to endogenous estrogen [15, 16]. Therefore, the risk of CVD among postmenopausal women with diabetics is higher. These findings are primarily based on studies conducted in Western populations. The relationship between the risk of CVD and menopause has been widely studied in China. Wu et al. [17] demonstrated that during a median follow-up period of 11.2 years, 246 out of 31,955 died from diabetes. In a Kailuan study, diabetic participants only accounted for 2.5% of all deaths [18]. A study by Gallagher et al. selected patients from among textile workers, but information on their diabetes status was not available [19]. These studies sampled the general population, but there is no research that has focused on diabetic patients in China [1, 17–19]. Therefore, the aim of the present study was to investigate the prevalence of CVD risk factors and assess the 10-year risk of CVD using the Framingham Risk Score (FRS) on 569 non-menopausal and postmenopausal women with type 2 diabetes mellitus in China.

## Methods

### Participants

This cross-sectional survey was performed at a tertiary hospital in Changzhou, Jiangsu Province, China, using a convenient sample size of 600 admission patients with type 2 diabetes mellitus between January 2016 and January 2018. The inclusion criteria were female patients aged 18 years or above, who were admitted to the Department of Endocrinology, and had type 2 diabetes mellitus for at least 3 months and were able to communicate fluently and clearly. Exclusion criteria were patients with type 1 diabetes mellitus, dementia or a severe illness, such as cancer or other immune system diseases, serious accompanied diseases or complications, psychosis, coronary heart disease, arrhythmia, stroke and those who were pregnant or lactating. After excluding 31 subjects with missing clinical characteristics, 569 out of the 600 eligible participants completed the surveys, representing a 95.5% response rate.

Patients who met the inclusion criteria and gave verbal informed consent were acquired to complete the surveys. The present study was approved by the Ethics Committee of the Hospital.

### Data collection

The patients were required to undergo a series of examinations, including demographic and clinical characteristics and a biochemical evaluation for the analysis. The information included age, diabetes course, family history, history of present illness, height, body weight, blood pressure, smoking, alcohol intake habits and current medications. Blood chemistry values assessed included HbA_1c_, plasma glucose, C peptide levels, 4 items of blood lipid tests and apolipoprotein concentrations. Blood sampling was performed at a clinical testing center of our hospital. Two health care professionals who were trained as data collectors used uniform instructions to guarantee the quality of data collection. Two other data collectors obtained further information on age at menarche, menstrual days and cycle, menopause age, treatment regimens, hypoglycemia and complications, through face to face interviews. We classified the 569 patients into non-menopause (*n* = 172), postmenopause < 5 y (*n* = 231) and postmenopause ≥5 y (*n* = 166) based on whether they were menopausal or not and time since menopause.

Patients were identified as dyslipidemic, if any one of the following indexes were met: total cholesterol (TC) level ≥ 240 mg/dL, low-density lipoprotein cholesterol (LDL-C) level ≥ 160 mg/dL, triglycerides (TG) level ≥ 88 mg/dL, high-density lipoprotein cholesterol (HDL-C) level ≤ 40 mg/dL or if the patients were taking lipid-regulating drugs [20].

A diagnosis of hypertension was made in the absence of antihypertensive drugs, when systolic blood pressure (SBP) ≥ 140 mmHg and/or diastolic blood pressure (DBP) ≥ 90 mmHg; a previous history of hypertension, currently using antihypertensive drugs [21]. Weight was classified as follows: normal weight, BMI < 24 kg/m^2^; overweight, BMI 24–27.9 kg/m^2^; Obese, BMI ≥ 28 kg/m^2^ [21].

A general check-up was performed by an internal medicine physician. Two independent radiologists interpreted the abdominal ultrasonography results of all patients at the time of the initial evaluation to determine the presence of NAFLD [22].

The target goals of blood glucose, blood pressure and blood lipids were HbA_1c_ < 7%, blood pressure < 130/80 mmHg and TC < 174 mg/dL, which are based on Guidelines for Diabetes Prevention and Treatment in China [21].

### Assessment of cardiovascular risk

FRS was used to evaluate the 10-year risk of CVD [23]. Based on the gender of the subjects, female FRS scores were selected and calculated using five risk factors, including age, TC, HDL-C, SBP and smoking. The cutoff values for calculating FRS are as follows: age, 20–34 y, − 7 points; 35–39 y, − 3 points; 40–44 y, 0 points; 45–49 y, 3 points; 50–54 y, 6 points; 55–59 y, 8 points; 60–64 y, 10 points; 65–69 y, 12 points; 70–74 y, 14 points; 75–79 y, 16 points; TC level < 160, 160–199, 200–239, 240–279, and ≥ 280 mg/dL; HDL-C level: < 40, 40–49, 50–59, and ≥ 60 mg/dL; SBP: < 120, 120–129, 130–139, 140–159, and ≥ 160 mmHg; nonsmoker or smoker [23]. The 10 year CVD risk percentage was calculated using the total number of points: < 9 points, < 1%; 9–12 points, 1%; 13–14 points, 2%; 15 points, 3%; 16 points, 4%; 17 points, 5%; 18 points, 6%; 19 points, 8%; 20 points, 11%, 21 points, 14%; 22 points, 17%; 23 points, 22%; 24 points, 27%; 25 points or more ≥30%. Absolute 10 year CVD risk percentage was classified as low risk, < 10%; moderate risk, 10–20%; and high risk, > 20% [23]. In the light of FRS, cardiovascular risk factors (age, TC, HDL-C, SBP and smoking) were assessed for all patients.

### Statistical analysis

IBM SPSS ver. 22.0 (SPSS Inc., Chicago, IL, USA) was used to perform the statistical analysis. All data are expressed as mean ± standard deviation or percentage, where applicable. T-test or one-way ANOVA (for continuous variables) and chi-squared test (for categorical variables) were used to compare data between different groups. Non-parametric tests, including Mann-Whitney U test and Kruskal-Wallis H test, were used for data that did not show a normal distribution. Spearman correlation was used to examine the relationship between 10-year risk of CVD and associated factors, which including age, diabetes course, complications, plasma glucose, body mass index, blood lipid profile, reproductive factors and treatment regimen. A stepwise multiple linear regression model was performed. The 10-year risk of CVD was the dependent variable. Independent variables were those parameters that were statistically correlated to 10-year risk of CVD. For all statistical tests, a *P* value of *<* 0.05 was considered to be statistically significant.

## Results

### Demographic and clinical characteristics among the three groups (*n* = 569)

The demographic and clinical characteristics of the study population and grouping are summarized in Table 1. The information included age (49.4 ± 8.1 y), diabetes course (5.2 ± 5.3 y), BMI (24.6 ± 5.1 kg/m^2^), waist-to-hip-ratio (0.904 ± 0.05), HbA_1c_ (9.6 ± 2.7%), FPG (9.6 ± 2.9 mmol/L), 2hPG (13.7 ± 4.0 mmol/L), SBP (134.1 ± 18.0 mmHg), DBP (83.2 ± 10.7 mmHg), TC (181.4 ± 38.6 mg/dL), TG (92.6 ± 81.1 mg/dL), HDL-C (42.5 ± 11.6 mg/dL), LDL-C (92.6 ± 30.9 mg/dL), Apo A1 (1.1 ± 0.19 g/L) and Apo B (1.0 ± 0.30 g/L) (Table 1). Reproductive factors included age at menarche (15.0 ± 1.0 y), menstrual days (5.2 ± 1.3 d) and menstrual cycle (30.0 ± 5.4 d) (Table 1). Statistically significant differences were found regarding age, diabetes course, BMI, menstrual days and menstrual cycle among non-menopause, postmenopause < 5 y and postmenopause ≥5 y subjects. Additionally, compared with postmenopause < 5 y patients, patients in the postmenopause ≥5 y group had a younger age at menopause, longer duration of time since menopause and shorter total number of reproductive years (i.e., the duration between menarche and menopause) (*P* <  0.001).

**Table 1:** Demographic and clinical characteristics of the non-menopause, postmenopause < 5 y and postmenopause ≥5 y patients (*N* = 569)

Variable	Total (*n* = 569)	Group			*P*-value*
		Non-menopause (*n* = 172)	Postmenopause< 5y (*n* = 231)	Postmenopause ≥5y (*n* = 166)	
Age, y	49.4 ± 8.1	40.7 ± 7.6^*b,c*^	51.2 ± 4.4^*a,c*^	56.0 ± 2.9^*a,b*^	< 0.001
Family history, %	276 (48.7)	86 (50.0)	110 (47.8)	80 (48.5)	0.910
Diabetes course, y	5.2 ± 5.3	3.5 ± 4.2 ^*b,c*^	5.1 ± 5.0 ^*a,c*^	7.1 ± 6.1 ^*a,b*^	< 0.001
FPG, mmol/L	9.6 ± 2.9	9.9 ± 2.9	9.7 ± 2.7	9.3 ± 2.9	0.173
2hPG, mmol/L	13.7 ± 4.0	14.0 ± 3.9	13.4 ± 3.9	13.9 ± 4.2	0.281
C peptide levels, nmol/L					
Fasting C peptide	1.9 ± 1.0	1.9 ± 1.2	1.8 ± 0.9	1.9 ± 0.9	0.781
30-min Postprandial C peptide	2.5 ± 1.4	2.5 ± 1.5	2.5 ± 1.3	2.6 ± 1.3	0.750
1-h Postprandial C peptide	3.4 ± 2.0	3.3 ± 2.0	3.3 ± 2.0	3.5 ± 1.9	0.482
2-h Postprandial C peptide	3.4 ± 2.5	3.0 ± 2.5	3.9 ± 2.5	4.2 ± 2.5	0.420
3-h Postprandial C peptide	3.3 ± 2.1	3.3 ± 2.1	3.3 ± 2.0	3.4 ± 2.1	0.663
BMI, kg/m^2^	24.6 ± 5.1	25.5 ± 3.6 ^*b,c*^	24.6 ± 3.5^*a*^	24.7 ± 3.3^*a*^	0.022
WC, cm	85.4 ± 9.1	85.7 ± 9.0	84.6 ± 8.8	86.1 ± 9.6	0.244
Waist-to-hip-ratio	0.904 ± 0.05	0.904 ± 0.04	0.901 ± 0.05	0.910 ± 0.05	0.216
SBP, mmHg	134.1 ± 18.0	132.9 ± 17.9	133.8 ± 17.8	135.9 ± 19.0	0.276
DBP, mmHg	83.2 ± 10.7	84.4 ± 12.1	83.3 ± 10.1	81.8 ± 10.7	0.232
TC, mg/dL	181.4 ± 38.6	181.4 ± 38.6	185.3 ± 38.6	181.4 ± 42.5	0.567
TG, mg/dL	92.6 ± 80.1	96.5 ± 88.8	92.6 ± 77.2	88.8 ± 77.2	0.672
HDL-C, mg/dL	42.5 ± 11.6	42.5 ± 11.6	42.5 ± 11.6	42.5 ± 11.6	0.304
LDL-C, mg/dL	92.6 ± 30.9	92.6 ± 27.0	96.5 ± 34.7	92.6 ± 30.9	0.742
Apo A1, g/L	1.1 ± 0.19	1.1 ± 0.15	1.1 ± 0.22	1.1 ± 0.18	0.632
Apo B, g/L	1.0 ± 0.30	1.0 ± 0.27	1.0 ± 0.21	1.0 ± 0.42	0.376
HbA_1c_, %	9.6 ± 2.7	9.8 ± 2.3	9.6 ± 3.2	9.4 ± 2.4	0.149
Complications, %					
Retinopathy	33 (5.8)	8 (4.7)	13 (5.6)	12 (7.2)	0.592
Neuropathy	122 (21.4)	25 (14.5)	47 (20.3)	50 (30.1)	0.002
Diabetic foot	5 (0.9)	0 (0.0)	2 (0.9)	3 (1.8)	0.205
Kidney disease	36 (6.3)	10 (5.8)	18 (7.8)	8 (4.8)	0.461
Ketoacidosis	73 (12.8)	35 (20.3)	30 (13.0)	8 (4.8)	< 0.001
Hypoglycemic agents, %	503 (88.6)	150 (87.2)	207 (89.6)	146 (88.5)	0.755
Insulin injections	358 (62.9)	118 (68.6)	139 (60.2)	101 (60.8)	0.179
Metformin	427 (75.0)	133 (77.3)	170 (73.6)	124 (74.7)	0.688
Sulfanylureas	120 (21.1)	32 (18.6)	54 (23.4)	34 (20.5)	0.496
Glinides	69 (12.1)	10 (5.8)	28 (12.1)	30 (20.1)	< 0.001
αglucosidase inhibitors	375 (65.9)	113 (65.7)	152 (65.8)	110 (66.3)	0.993
Pioglitazone	37 (6.5)	13 (7.6)	16 (6.9)	8 (4.8)	0.561
Insulin plus oral medication	314 (55.2)	94 (54.7)	133 (57.6)	87 (52.4)	0.706
Treatment regimens, %					
Antihypertensive agents	170 (78.3)	29 (13.4)	82 (82.8)	59 (78.7)	0.123
Lipid modulators	103 (50.0)	25 (39.7)	49 (55.7)	29 (52.7)	0.137
Antithrombotic drugs	156 (27.1)	24 (14.0)	79 (34.2)	53 (31.9)	< 0.001
Alcohol intake, %	2 (0.4)	0	1 (0.4)	1 (0.6)	0.622
Hypoglycemia, %	39 (6.9)	5(2.9)	22 (9.5)	12 (7.2)	0.033
Reproductive factors					
Age at menarche, y	15.0 ± 1.0	14.9 ± 1.1	15.1 ± 0.9	15.1 ± 1.1	0.140
Menstrual days, day	5.2 ± 1.3	5.4 ± 1.3^*c*^	5.1 ± 1.3	5.0 ± 1.3^*a*^	0.048
Menstrual cycle, day	30.0 ± 5.4	31.3 ± 8.5^*b,c*^	29.6 ± 3.6^*a*^	29.3 ± 1.9^*a*^	0.010
Reproductive years, y	–	–	34.5 ± 4.0	32.8 ± 3.7	< 0.001
Menopause age, y			49.6 ± 3.9	47.9 ± 3.5	< 0.001
Time since menopause, y	–	–	1.66 ± 1.58	8.2 ± 2.7	< 0.001

^*a*^Compared with the non-menopausal group, *P* <  0.05;

^*b*^Compared with the postmenopause < 5 y group, *P* <  0.05;

^*c*^Compared with the postmenopause ≥5 y group, *P* <  0.05;

^*^Compared among groups

### Prevalence of the cardiovascular risk factors among all subjects

Among the T2DM inpatients, the exposure rate of NAFLD, overweight or obesity, hypertension, dyslipidemia and smoking was 58.2, 56.6, 38.1, 36.2 and 0.7%, respectively. The prevalence of hypertension tended to increase with age (Table 2). The usage rate of hypoglycemic agents (including insulin injections and oral medication), antihypertensive agents, lipid modulators and antithrombotic drugs was 88.6, 78.3, 50.0 and 27.1%, respectively (Table 1). Only 1.2% of patients achieved all 3 target goals i.e. blood glucose (HbA_1c_ < 7%), blood pressure (systolic blood pressure < 130 mmHg and diastolic blood pressure < 80 mmHg), and blood lipids (total cholesterol < 174 mg/dL) [21].

**Table 2:** The distribution of cardiovascular risk factors among the different groups (%)

Group	n	Hypertension	Dyslipidemia	NAFLD	Overweight or obesity	Smoking
Non-menopause	172	43 (25.0)	63 (36.6)	105 (61.0)	109(63.4)	1 (0.6)
Postmenopause<5y	231	99 (42.9)	88 (38.1)	131 (56.7)	123 (53.2)	3 (1.3)
Postmenopause ≥5y	166	75 (45.2)	55 (33.1)	95 (57.2)	90 (54.2)	0
*x*^*2*^		18.254	1.049	0.848	7.343	2.386
*P*		< 0.001	0.592	0.655	0.119	0.303

### Framingham risk score and 10-year risk of CVD among the three groups

The Framingham Risk Score was (10.3 ± 4.5), and the 10-year risk of CVD was (1.6 ± 1.5%), and both indicator values increased along with the age (*P* <  0.001) (Table 3).

**Table 3:** Framingham Risk Score and 10-year risk of CVD

Group	n	Framingham Risk Score	10-year risk of CVD (%)
Non-menopause	172	6.3 ± 4.5^b,c^	1.0 ± 0.4 ^b,c^
Postmenopause < 5y	231	11.3 ± 3.4^a,c^	1.7 ± 1.3 ^a,c^
Postmenopause ≥5y	166	12.9 ± 2.5^a,b^	2.1 ± 2.0 ^a,b^
*F*		163.357	27.726
P^*^		< 0.001	< 0.001

^a^Compared with the non-menopausal group, *P* <  0.05;

^b^Compared with the postmenopause <5 y group, *P* <  0.05;

^c^Compared with the postmenopause ≥5 y group, *P* <  0.05;

^*^Compared among groups

### Related factors associated with 10-year risk of CVD among all patients

Among all subjects, the 10-year risk of CVD was a dependent variable, in addition to age, TC, SBP, smoking, diabetes course, complications, FPG, 2hPG, BMI, waist-to-hip-ratio, TG, LDL-C, Apo A_1_, Apo B and menopausal state (no = 0, yes = 1), while the treatment regimens were independent variables, which were performed in a stepwise multiple linear regression model. The results are shown in Table 4. The 10-year risk of CVD was found to be positively correlated with menopausal state (no = 0, yes = 1), LDL-C, FPG and waist-to-hip-ratio (*β* = 0.275, 0.212, 0.093, − 0.078, respectively, *P* < 0.05), which may explain the variance of 14.3%. Menopause, LDL-C, FPG and waist-to-hip-ratio were found to be independent risk factors for the 10-year risk of CVD.

**Table 4:** Stepwise multiple linear regression for 10-year risk of CVD and related factors among all subjects (*N* = 569)

Model	Unstandardized coefficients *B*	Standardized coefficients *β*	*t*	*P*-value
Menopause or not	0.868	0.275	7.043	< 0.001
LDL-C	0.387	0.212	5.379	< 0.001
FPG	0.046	0.093	2.364	0.018
Waist-to-hip-ratio	2.238	0.078	1.993	0.047
Constant	−3.249	–	−3.104	0.002

*R*^*2*^ = 0.143

### Risk factors associated with 10-year risk of CVD in postmenopausal patients

In the postmenopausal group, 10-year risk of CVD was as dependent variable. Except for age, TC, SBP and smoking, the independent variables were diabetes course, complications, FPG, 2hPG, BMI, waist-to-hip-ratio, TG, LDL-C, Apo A_1_, Apo B, menopause age, time since menopause, total reproductive years and treatment regimens. LDL-C (*β* = 0.227, *P* < 0.001), FPG (*β* = 0.139, *P* = 0.003) and time since menopause (*β* = 0.230, *P* < 0.001) were found to be associated with CVD, which may explain the variance of 14.6% (Table 5).

**Table 5:** Stepwise multiple linear regression for 10-year risk of CVD and related factors in postmenopausal patients (*n* = 397)

Model	Unstandardized coefficients *B*	Standardized coefficients *β*	*t*	*P*-value
LDL-C	0.449	0.227	4.783	< 0.001
FPG	0.078	0.139	2.947	0.003
Time since menopause	0.099	0.230	4.672	< 0.001
Constant	−4.568	–	−4.069	< 0.001

*R*^*2*^ = 0. 146

## Discussion

The findings of the present study indicate that the incidence of dyslipidemia, hypertension, overweight or obesity and NAFLD is high. All these indicators are considered to be conventional cardiovascular risk factors. The association between these factors and cardiovascular disease is well known [21, 24–26]. The prevalence of dyslipidemia, hypertension, overweight or obesity and NAFLD in diabetic patients was found to be 36.2, 38.1, 56.6 and 58.2%, respectively, in the current study, which is higher than the rates reported of Ma et al. [27], Bachir Cherif et al. [28], Echouffo-Tcheugui et al. [29] and Younossi et al. [30]. The total prevalence in their studies were found to be 13.1, 31.6, 44.5 and 25.24%, respectively. The explanation for these differences may be due to differences among sample populations. In our study, subjects were recruited from among inpatients who had suffered from diabetes under critical conditions. The participants of the other studies were selected from a community dwelling or were outpatients, and most of them were healthy. In the current study, except for hypoglycemic agents, the usage rate of antihypertensive agents, lipid modulators and antithrombotic drugs was low. According to the guidelines for T2DM, health care professionals should promptly detect T2DM patients with hypertension, dyslipidemia and NAFLD, and provide early treatment measures, such as antihypertensive agents, lipid modulators and aspirin [21]. However, the treatment status of these Chinese T2DM inpatients was not ideal. In addition, the target goals for the control of blood glucose, blood pressure and blood lipids was only 1.2%, which is significantly lower than the 5.6% reported among type 2 diabetic outpatients [31]. Thus, achieving adequate control of risk factors for cardiovascular disease in patients with type 2 diabetes remains a clinical challenge [31]. For diabetic patients, complications may include dyslipidemia, hypertension, overweight or obesity, NAFLD. Therefore, healthcare professionals should not only focus on helping patients monitor their plasma glucose levels, but also intervene to achieve control of blood pressure and serum lipid.

We found that a higher risk of CVD was observed among diabetic patients who have experienced menopause, compared with non-menopausal women. The related factors are LDL-C and FPG levels. Two studies have shown that menopause has a marked effect on the circulation levels of lipids and lipoproteins, with an especially significant increase in LDL-C levels [32, 33]. Previous studies have examined the association between FPG, 2hPG and CVD risk in a community [18, 34, 35]. These studies have reported significant results, which are consistent with our findings. Shen et al. also indicate that postmenopausal women show significant association with worse glycemic control, including FPG levels, which is independently associated with 10-year cardiovascular risk [36, 37]. Additionally, diabetes is associated with chronic inflammation, which is characterized by the release of excess pro-inflammatory cytokines, abrupt levels of acute-phase proteins, and other mediators, which are integral to the severity of cardiovascular disorders [38–40]. Menopause complicated with diabetes is an independent predictor of cardiovascular disease. Our study shows that the waist-to-hip-ratio is a protective factor for 10-year risk of CVD. In a study conducted on five hundred postmenopausal women study, truncal obesity with a waist-hip ratio > 0.8 was found in 68% of females, which does not indicate the association between waist-to-hip ratio and 10-year risk of CVD [41]. This is an urgent matter for healthcare providers to assist these patients in establishing a good lifestyle, in order to maintain a proper waist-to-hip ratio.

Furthermore, as the period of time since menopause increases, the CVD risk also increases. In a Chinese population study, compared to women who had been menopausal for less than 1 year, those with elapsed time since menopause of 2–3 years had higher coronary heart disease prevalence, and higher TG levels [42]. Cho et al. also determined that 10 to 14 years after menopause, postmenopausal women show an increased risk of high TG [43]. Additionally, time since menopause may related to some conventional CVD risk factors, such as metabolic syndrome or obesity [40, 43]. But a review has shown that there is no meaningful relationship that has emerged between time since menopause and CVD risk factors [44]. Further, a study of the relationship between time since menopause and CVD risk or relevant diseases is needed to explain the biological mechanisms.

This study has several limitations. First, although this is the first study to report that cardiovascular risk factors of menopausal women with type 2 diabetes mellitus, the study used a hospital-based cohort of a Chinese population, indicating that the study sample was from a single hospital. We cannot assume that our findings can be generalize to other groups. Second, the study population was hospitalized patients, which may have introduced a selection bias. In addition, there was also a memory bias in the study, especially when we asked some questions about reproductive factors, such as menstrual days, menstrual cycle, menopausal age, complications, and treatment regimen, subjects had to recall what had happened in the past, which is not the best method. Third, the lack of a non-diabetic age-matched control group was also a limitation of this study. Fourth, due to the observational nature of the study, the mechanisms proposed to explain the association between higher risk of CVD, menopausal status and FPG are merely speculative. Indeed, no tests of sex hormones have been performed. Finally, an analysis of the difference in autoantibody positivity between the non-menopause group and the menopause groups was not conducted, which is a shortcoming of this study.

## Conclusions

In conclusion, among women with type 2 diabetes mellitus in China, the prevalence of cardiovascular risk factors are high. Although there is overwhelming evidence to indicate that achieving adequate control of blood pressure and lipids can significantly delay or prevent the onset of CVD, successful accomplishment of recommended therapeutic goals is a huge challenge. In diabetic females, except for menopausal state and time since menopause being uncontrollable factors, LDL-C, FPG and waist-to-hip ratio were found to be controllable factors. The clinical management of diabetes needs change. In addition to monitoring plasma glucose, interventions to improve blood pressure, serum lipids and lifestyle are needed. These women are currently too young to have experienced CVD events, therefore, the continued follow-up of these women needs to be conducted.

## Data Availability

The datasets used and/or analyzed during the current study are available from the corresponding author on reasonable request.

## Abbreviations

2hPG: 2 h postprandial glucose
Apo A1: Apolipoprotein A1
Apo B: Apolipoprotein B
BMI: Body mass index
CVD: Cardiovascular disease
DBP: Diastolic blood pressure
FPG: Fasting plasma glucose
FRS: Framingham risk score
HbA_1c_: Hemoglobin A_1c_
HDL-C: High-density lipoprotein cholesterol
LDC-C: Low-density lipoprotein cholesterol
NAFLD: Nonalcoholic fatty liver disease
SBP: Systolic blood pressure
T2DM: Type 2 diabetes mellitus
TC: Total cholesterol
TG: Triglycerides
WC: Waist circumference
